# Wind-Speed-Adaptive Resonant Piezoelectric Energy Harvester for Offshore Wind Energy Collection

**DOI:** 10.3390/s24051371

**Published:** 2024-02-20

**Authors:** Weijian Wu, Zhen Pan, Jiangtao Zhou, Yingting Wang, Jijie Ma, Jianping Li, Yili Hu, Jianming Wen, Xiaolin Wang

**Affiliations:** 1 The Institute of Precision Machinery and Smart Structure, College of Engineering, Zhejiang Normal University, Yingbin Street 688, Jinhua 321004, China; wuweijian@zjnu.edu.cn (W.W.); pz72988@zjnu.edu.cn (Z.P.); zhoujiangtao@zjnu.edu.cn (J.Z.); wangyingting@zjnu.edu.cn (Y.W.); mjj@zjnu.cn (J.M.); lijp@zjnu.cn (J.L.); huyili@zjnu.edu.cn (Y.H.); wjming@zjnu.cn (J.W.); 2 Key Laboratory of Intelligent Operation and Maintenance Technology & Equipment for Urban Rail Transit of Zhejiang Province, Zhejiang Normal University, Yingbin Street 688, Jinhua 321004, China

**Keywords:** piezoelectric energy harvester, offshore winds, adaptive, sub-resonant, coil spring

## Abstract

This paper proposes a wind-speed-adaptive resonant piezoelectric energy harvester for offshore wind energy collection (A-PEH). The device incorporates a coil spring structure, which sets the maximum threshold of the output rotational frequency, allowing the A-PEH to maintain a stable output rotational frequency over a broader range of wind speeds. When the maximum output excitation frequency of the A-PEH falls within the sub-resonant range of the piezoelectric beam, the device becomes wind-speed-adaptive, enabling it to operate in a sub-resonant state over a wider range of wind speeds. Offshore winds exhibit an annual average speed exceeding 5.5 m/s with significant variability. Drawing from the characteristics of offshore winds, a prototype of the A-PEH was fabricated. The experimental findings reveal that in wind speed environments, the device has a startup wind speed of 4 m/s, and operates in a sub-resonant state when the wind speed exceeds 6 m/s. At this point, the A-PEH achieves a maximum open-circuit voltage of 40 V and an average power of 0.64 mW. The wind-speed-adaptive capability of the A-PEH enhances its ability to harness offshore wind energy, showcasing its potential applications in offshore wind environments.

## 1. Introduction

To achieve carbon emission reduction goals, countries worldwide are actively seeking sustainable energy alternatives to replace traditional fossil fuels [1]. Maximizing the utilization of offshore wind energy stands as an effective pathway towards achieving lower carbon emissions [2]. Offshore winds typically boast a higher energy density, with an annual average wind speed exceeding 5.5 m/s [3]. Effectively harnessing offshore wind energy can significantly reduce the reliance on conventional energy sources. Among the common mechanisms for wind energy collection are electromagnetic induction [4], a tribo-electric mechanism [5], and a piezoelectric mechanism [6]. Piezoelectric energy harvesters have captured significant attention among researchers due to their advantageous features of a simple structure, high power density, and cost-effectiveness.

Piezoelectric energy harvesters can be broadly classified into two main categories: non-resonant and resonant types, based on their operational characteristics. In the realm of fluid energy harvesting, non-resonant piezoelectric energy harvesters employing broadband techniques can be categorized, based on the principles of fluid–structure coupling, into galloping piezoelectric energy harvesters [7,8,9,10] and fluttering piezoelectric energy harvesters [11,12,13,14]. For galloping piezoelectric energy harvesters, Zhao et al. [15] proposed a funnel-shaped galloping energy harvester with a wide working wind speed range and high normalized harvesting power. Wang et al. [16] proposed a novel tristable galloping-based piezoelectric energy harvester by introducing a nonlinear magnetic force. For fluttering piezoelectric energy harvesters, Li et al. [17] introduced a hybrid piezoelectromagnetic energy harvester designed for vehicles, aiming to capture and harness both the wind and vibration energy generated during driving. Cheng et al. [18] proposed an innovative design of a cantilevered piezoelectric generator with airfoil-based structures for harvesting underutilized aeroelastic energy in the pneumatic system. Non-resonant piezoelectric energy harvesters can function across a broader range of wind speeds, they face challenges with a high startup wind speed, and their structures are susceptible to damage when the wind speed surpasses the bandwidth limit. While the overall output performance of non-resonant piezoelectric energy harvesters improves, the instantaneous output performance is lower than that during resonance.

Resonant piezoelectric energy harvesters demonstrate resonance at specific wind speeds, showcasing a remarkable output performance. Resonant piezoelectric energy harvesters commonly encompass vortex-induced piezoelectric energy harvesters [19,20,21,22] and rotary piezoelectric energy harvesters [23,24,25,26]. For vortex-induced piezoelectric energy harvesters, Zhang et al. [27] proposed an operable strategy to enhance the output power of piezoelectric energy harvesting from vortex-induced vibration using nonlinear magnetic forces. Meng et al. [28] presented an idea of employing a sphere as a bluff body subjected to cross flows for improving piezoelectric energy harvesting from flow-induced vibrations (FIVs). For rotary piezoelectric energy harvesters, Yu et al. [29] proposed a nonlinear sickle-shaped cantilever beam piezoelectric energy harvester (S-PEH) for wind energy harvesting. Zhang. et al. [30] proposed an axially retractable bracket-shaped piezoelectric vibrator excited by a magnetic force to provide a practical solution to the low reliability and narrow bandwidth of the traditional turbine-based piezoelectric wind energy harvesters (PWEHs). Resonant piezoelectric energy harvesters are well suited for low wind speed environments and offer robustness for piezoelectric elements during operation. However, their narrow operational wind speed range, dictated by the necessity for the resonance frequency to achieve a high output performance, limits their adaptability to the wide fluctuations in natural wind speeds. Therefore, a novel approach is required to enable resonant piezoelectric energy harvesters to sustain a sub-resonant operational state across a broad range of wind speeds, thereby enhancing their adaptability to varying wind conditions.

This paper proposes a wind-speed-adaptive resonant piezoelectric energy harvester for offshore wind energy collection. The A-PEH includes an energy harvesting module, a frequency modulation (FM) module, and a power generation module. The frequency modulation module, utilizing a unique coil spring structure, adjusts the device’s output rotational frequency when the wind speed exceeds a preset threshold. Therefore, the A-PEH is wind-speed-adaptive, allowing it to sustain a sub-resonant state across a broad spectrum of wind speeds. Additionally, the A-PEH features lower startup wind speeds and an extended lifespan. Experimental validation in this study confirms the A-PEH’s capability to harvest wind energy within the wind speed range encountered offshore.

## 2. Theoretical Principles

### 2.1. Structural Design

**Figure 1:**
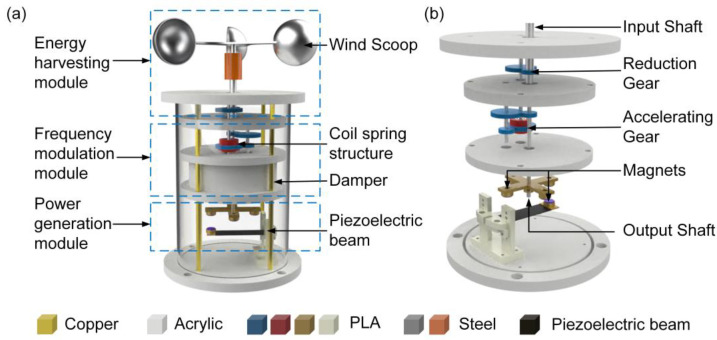
Schematic diagrams of the A-PEH: (**a**) Overall structure of the A-PEH. (**b**) Mechanical transmission part.

The structure of the A-PEH is shown in Figure 1a. The A-PEH consists of an energy harvesting module for wind energy collection, a frequency modulation (FM) module to regulate the output rotational frequency, and a power generation module for converting mechanical energy into electrical energy. The energy harvesting module consists of a wind cup and reduction gears, while the FM module includes a coil spring structure and a damper. The power generation module comprises a piezoelectric beam and magnets. Two sets of speed-enhancing gears are positioned between the FM module and the power generation module. The mechanical energy transfer component is depicted in Figure 1b. The energy conversion process begins with the wind cups transforming wind energy into mechanical energy, which is then transferred to the input shaft. Subsequently, the input shaft transmits the energy to the coil spring via a set of reduction gears with a reduction ratio of 2:1. The coil spring then transfers the energy to the output shaft through two sets of speed-up gears, featuring a gear ratio of 1:2. Ultimately, the output shaft rotates the magnets to drive the power generation module. The mechanical energy transfer section increases the rotational frequency of the input shaft by a factor of 8, delivering it as the excitation frequency for the power generation module.

### 2.2. Working Principle

**Figure 2:**
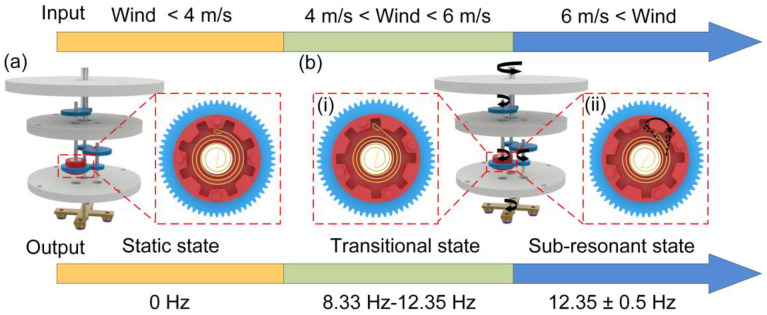
Operating principle of the A-PEH. (**a**) Schematic diagram of A-PEH in static state. (**b**) A-PEH is in transitional state and sub-resonant state.

Figure 2 illustrates the operational principle of the A-PEH in achieving wind-speed adaptivity. The A-PEH exhibits three distinct operational states under varying wind speeds: a static state, transitional state, and sub-resonant state.

In wind speeds below 4 m/s, the torque from the wind is insufficient to overcome the resistance torque of the A-PEH, resulting in a static state. The coil spring remains static in this condition, as shown in Figure 2a. The A-PEH undergoes a transitional state when wind speeds range between 4 and 6 m/s. At this point, the resistance torque of the A-PEH is less than the torque provided by the wind. Throughout this period, the output performance of the A-PEH improves with the increasing wind speed, and the excitation frequency of the power generation module ranges from 8.33 Hz to 12.35 Hz. Upon reaching a wind speed of 6 m/s, the A-PEH enters a sub-resonant state, with the excitation frequency of the power generation module hovering around 12.35 Hz. The contracted state of the coil spring characterizes this phase, as depicted in Figure 2(bi). In both the static and transitional states, the coil spring merely serves as a mechanical connector. Beyond a wind speed of 6 m/s, the coil spring transitions into an intermittent energy release state, as illustrated in Figure 2(bii). The intermittent energy release state of the coil spring is pivotal for the A-PEH to achieve wind-speed adaptivity. When the external wind speed increases and the input torque exceeds the maximum torque of the coil spring, the diameter of the coil spring becomes smaller than the inner diameter of the coil spring mounting structure, causing the coil spring to disengage. During the disengagement of the coil spring, excess energy stored within it is released, causing its diameter to return to the same as the inner diameter of the coil spring mounting structure, and leading to the reengagement of the coil spring with the mounting structure under the maximum torque. A disengagement–reengagement process constitutes an energy release cycle of the intermittent energy release state of the coil spring, which can be completed in a short time. Consequently, when the coil spring enters the intermittent energy release state, the coil spring mounting structure can be approximated as constantly driven by the maximum torque of the coil spring. This allows the device’s excitation frequency to remain within the sub-resonant range of the piezoelectric beam. The intermittent energy release state of the coil spring is triggered when the wind speed exceeds the predetermined threshold, enabling the device to be wind-speed-adaptive when the wind speed exceeds this threshold.

**Figure 3:**
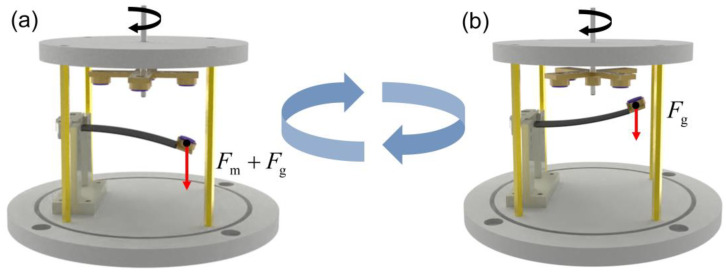
Working principle of the power generation module. (**a**) The piezoelectric beam vibrates downward. (**b**) The piezoelectric beam vibrates upward.

The working principle of the power generation module is depicted in Figure 3, where *F*_g_ represents the gravitational force on the tip mass and *F*_m_ signifies the magnetic force. During the A-PEH’s operation, the output shaft induces the rotation of magnets, utilizing the magnetic force to induce vibration in the piezoelectric beam. The four magnets on the output shaft are arranged in a circular array. When the center of the magnet on the piezoelectric beam aligns with the center of the rotating magnets, the piezoelectric beam vibrates downward under the influence of the magnetic force, as depicted in Figure 3a. Subsequently, as portrayed in Figure 3b, the magnetic force acting on the piezoelectric beam diminishes when the center of the magnet on the piezoelectric beam deviates from the same axis as the center of the rotating magnets. Consequently, the piezoelectric beam vibrates upward due to the effect of its own restoring force. The rotation of the output shaft induces periodic vibration in the piezoelectric beam, generating an alternating current.

### 2.3. Theory Analysis

**Figure 4:**
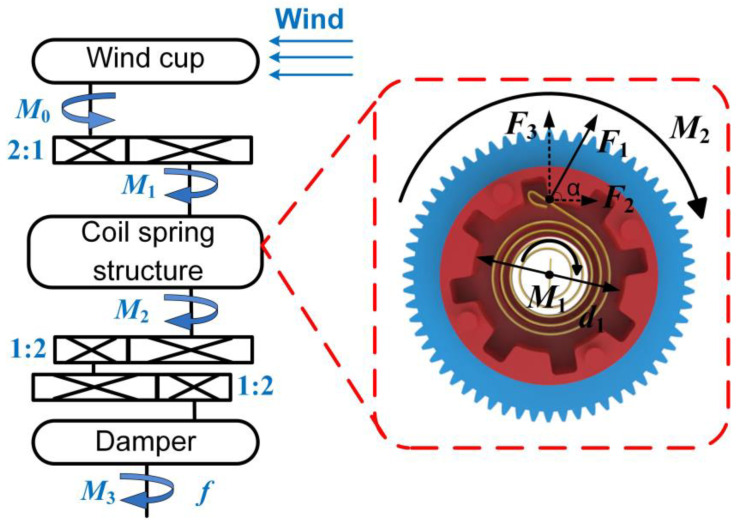
Schematic diagram of the transmission structure of A-PEH.

The schematic diagram of the transmission structure of the A-PEH is depicted in Figure 4. The input torque exerted on the wind cup by the wind is denoted as *M*_0_.

(1) $$M_{1}=2M_{0}$$

where *M*_1_ is the torque on the transmission shaft connecting to the coil spring.

The stiffness of the coil spring can be calculated using the following equation [31].

(2) $$k=\frac{Ebt^{3}}{12L}$$

where *E* is the Young’s modulus, *b* is the width of the spring strip, *t* is the thickness of a coil spring, and *L* is the length of the strip.

The force exerted by the coil spring on the mounting structure is denoted as *F*_1_, where *F*_2_ and *F*_3_ represent the tangential and radial components of the spring force. The free coil length *L* of the coil spring determines its initial diameter *d*_2_. The variation in the angular displacement of the coil spring correlates linearly with changes in the torque and free length [32]. Consequently, the reduction in the coil spring diameter shows a linear relationship with the torque. The maximum torque *M*_max_ exerted by the coil spring within the spring structure can be computed.

(3) $$M_{\max}=\beta k(d_{2}-d_{1})\cos\alpha d_{1}$$

where *β* is the correlation coefficient between the variation in the coil spring diameter and the angular displacement of the coil spring, *d*_1_ is the inner diameter of the coil spring mounting structure, and α is the angle between the direction of the coil spring force and the tangential direction.

When the input torque *M*_1_ is less than *M*_max_, the coil spring acts as a mechanical connector, and the output torque of the coil spring structure is *M*_2t_. When the input torque *M*_1_ exceeds *M*_max_, the coil spring enters a state of intermittent energy release, and the output torque of the coil spring structure is *M*_2r_.

(4) $$\left\{\begin{array}{ll} M_{2t}=M_{1} & \left(M_{1}<M_{\max}\right)\\ M_{2r}=M_{\max} & \left(M_{1}\geq M_{\max}\right) \end{array}\right.$$

The damper selected is a centrifugal friction damper. With a constant wind speed, the fluctuation in the output shaft speed is minimal, approximating uniform rotation. The resistance torque of the damper is *M*_f_.

(5) $$M_{f}=4\pi^{2}f^{2}r^{2}\mu N$$

where *f* is the rotational frequency of the device output, *r* is the inner diameter of the damper stator, *μ* is the friction coefficient, and *N* is the number of friction pads.

When the device is in the transitional state, the final output torque of the device is *M*_3t_. When the device is in the sub-resonant state, the final output torque of the device is *M*_3r_.

(6) $$\left\{\begin{array}{ll} M_{3t}=\frac{1}{2}M_{0}-M_{f} & \left(M_{1}<M_{\max}\right)\\ M_{3r}=\frac{1}{4}M_{\max}-M_{f} & \left(M_{1}\geq M_{\max}\right) \end{array}\right.$$

The coil spring structure renders the device wind-speed-adaptive, ensuring a stable output torque when the wind speed exceeds the predetermined threshold.

### 2.4. Fabrication of the A-PEH

**Figure 5:**
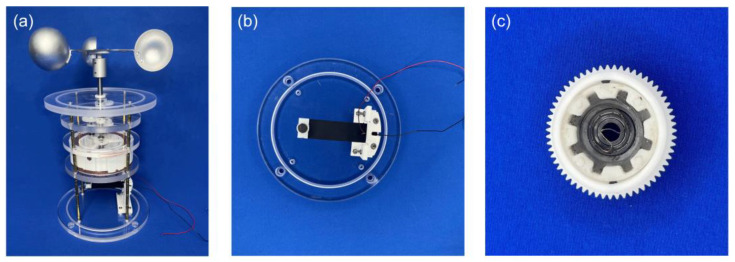
Photograph of the A-PEH. (**a**) Overall device. (**b**) Piezoelectric beam. (**c**) Coil spring structure.

The dimensions of the wind-speed-adaptive resonant piezoelectric energy harvester (A-PEH) are 166 mm (diameter) × 210 mm (height). The fixing plate of the A-PEH is manufactured using fine engraving and the material is acrylic. The transmission gears, coil spring outer barrel, piezoelectric beam fixtures, and magnet fixtures are made by 3D printing technology. The printing material is polylactic acid (PLA). The dimensions of the coil spring are 2 mm (width) × 0.1 mm (thickness). The magnet is a neodymium magnet with dimensions of 10 mm (diameter) × 2.5 mm (height). The size of the piezo beam is 80 mm × 20 mm × 0.2 mm, and the material of the piezoelectric transducer is PZT, with dimensions measuring 60 mm × 20 mm × 0.2 mm. The overall physical diagram of the A-PEH is shown in Figure 5a, the piezoelectric beam and tip magnet are shown in Figure 5b, and the coil spring structure is shown in Figure 5c.

## 3. Experimental Section

**Figure 6:**
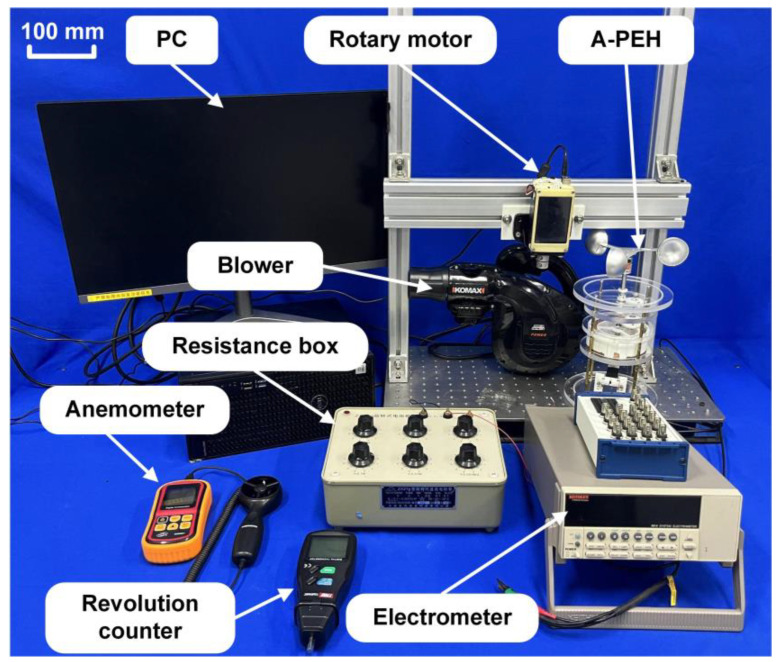
Test system of the A-PEH.

A test system for the A-PEH is shown in Figure 6. The testing system comprises a PC, rotary motor, blower, A-PEH, resistance box, anemometer, revolution counter, and electrometer. External excitation is provided by a blower (GPG-08SC, Komax, Shanghai, China) and a rotating motor (GPG-08SC, GPG Gearhead, Wenzhou, China). The blower modulates the airflow to simulate the different wind speeds found in nature, while the rotary motor varies the input rotational frequency of the device to replicate the various wind speeds encountered in natural environments. A programmable electrometer (6514, Keithley, Cleveland, OH, USA) and a data acquisition system (PCI-6259, NationalInstruments, Louisville, CO, USA) are used to measure and acquire the output signal of the A-PEH. The rotational speed sensor (TA8146C, TASI, Suzhou, China) is used to measure the rotation frequency of the input shaft and the anemometer is used to measure the wind speed at the wind cup (GM8901, BENETECH, Shenzhen, China). The collected signals are recorded by the LabVIEW program. During the experiment, the output performance of the A-PEH is assessed using the device’s open-circuit voltage and average power. The laboratory is located at Zhejiang Normal University, 688 Yingbin Street, Jinhua, Zhejiang, China. The testing was conducted in August 2023.

## 4. Results and Discussion

### 4.1. Parameter Optimization

**Figure 7:**
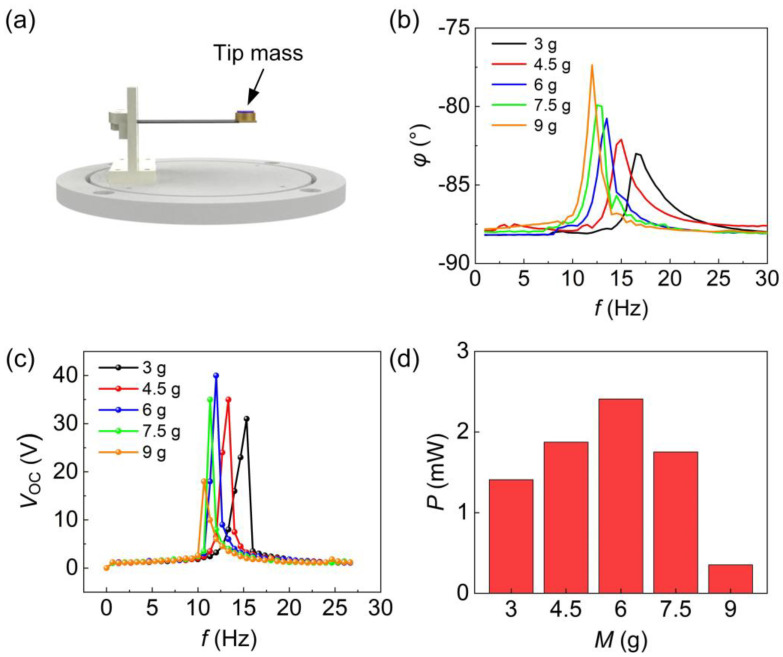
Piezoelectric beam tip mass optimization. (**a**) Tip mass position. (**b**) Phase angle versus frequency curves for PEH at different tip masses. (**c**) PEH open-circuit voltage versus excitation frequency curves for different tip masses. (**d**) Peak output power of PEH with different tip masses at an external load of 30 kΩ.

The effect of the tip mass of the piezoelectric beam on its output performance was investigated, as shown in Figure 7. The position of the tip mass is indicated in Figure 7a. The resonant frequency of the piezoelectric beam decreases with an increase in the tip mass [33]. As per prior research, the initial selection for the tip mass of the piezoelectric beam includes 3 g, 4.5 g, 6 g, 7.5 g, and 9 g. The piezoelectric beam’s resonant frequency, signified by the frequency at which the beam’s phase angle experiences an abrupt change during the sweep, is determined through an impedance analyzer for various tip masses. The resonant frequencies of the piezoelectric beam at tip masses of 3 g, 4.5 g, 6 g, 7.5 g, and 9 g are observable from Figure 7b. Figure 7c presents the output performance of piezoelectric beams with different tip masses at varying input frequencies. When the tip mass of the piezoelectric beam is 6 g, the open-circuit voltage reaches a maximum of 40 V at the sub-resonant state, occurring at an excitation frequency of 12 Hz. While a 6 g tip mass yields the maximum open-circuit voltage, it is crucial to consider that the resonant frequency is relatively low and may not necessarily result in achieving the maximum output performance. As illustrated in Figure 7d, the effective output power of the piezoelectric beams was tested under an external load of 30 kΩ to determine the maximum output performance of the piezoelectric beams with varying tip masses. The piezoelectric beam attains its highest output performance with a tip mass of 6 g, evidenced by a peak output power of 2.42 mW at the sub-resonant state. Consequently, it is deduced that the piezoelectric beam exhibits the optimal output performance with a tip mass of 6 g.

**Figure 8:**
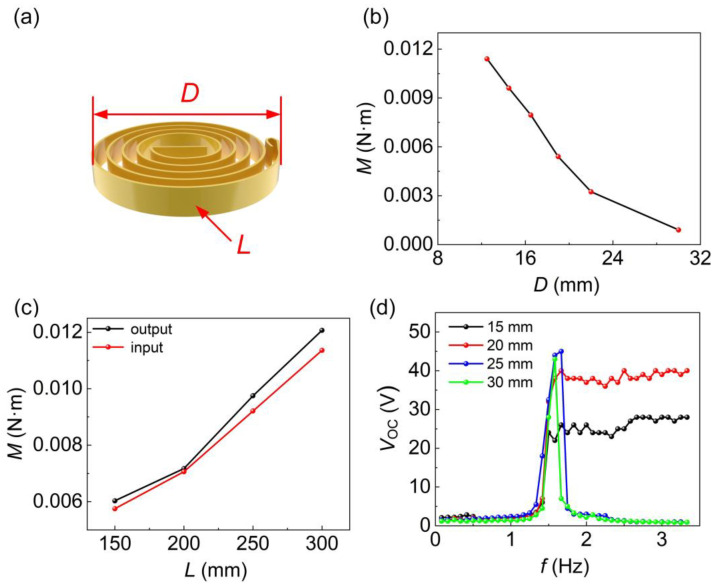
Coil spring length optimization. (**a**) Coil spring model. (**b**) Torque curve versus diameter. (**c**) The maximum input and output torque of coil springs of different lengths. (**d**) Variation curves of open-circuit voltage versus input rotational frequency for A-PEH with different lengths of coil springs.

The parameter of the coil spring plays a crucial role in determining the threshold speed of the FM module. Figure 8a depicts the schematic diagram of the coil spring, where *D* represents the diameter and *L* represents the length. The correlation between the torque of a coil spring and the compression of its diameter is illustrated in Figure 8b. As the diameter of the coil spring is compressed from 32 mm to 12.5 mm, the compression of the spring diameter exhibits a direct proportional increase in the spring moment. Due to the significant measurement errors in the diameter of the coil spring, subsequent tests were conducted using the length of the coil spring as the variable. Testing of the torque was conducted using four different lengths of spiral springs: 15 mm, 20 mm, 25 mm, and 30 mm. During the test, the input torque gradually increases until tripping occurs. At this point, the input torque on the shaft and the maximum output torque on the gear of the coil spring structure were measured. The analysis of Figure 8c reveals a linear relationship between the input torque and the maximum output torque concerning the length of the coil spring. Figure 8d illustrates the correlation between the open-circuit voltage of the A-PEH and the changes in the input rotational frequency while using coil springs of varying lengths. When the length of the coil springs are 25 mm and 30 mm, the torque required for the compression of the coil spring is excessively high, preventing it from entering the energy release state. Consequently, the device can only enter a sub-resonant state within a limited range of input rotational frequencies and is unable to adapt to a wider range of input rotational frequencies. When the coil spring length is 15 mm, the device can maintain a constant output rotational frequency when the input rotational frequency exceeds the threshold, achieving adaptability to the input rotational frequency. However, due to the low threshold frequency, the piezoelectric beam fails to reach the sub-resonant state, resulting in the reduced output performance of the device. When the coil spring length is 20 mm, the device can exhibit adaptability to the input rotational frequency. Additionally, with the appropriate threshold frequency, the piezoelectric beam can enter a sub-resonant state over a wider range of input frequencies, resulting in the generation of a 40 V open-circuit voltage. The optimal length of the coil spring is 20 mm.

### 4.2. Performance

**Figure 9:**
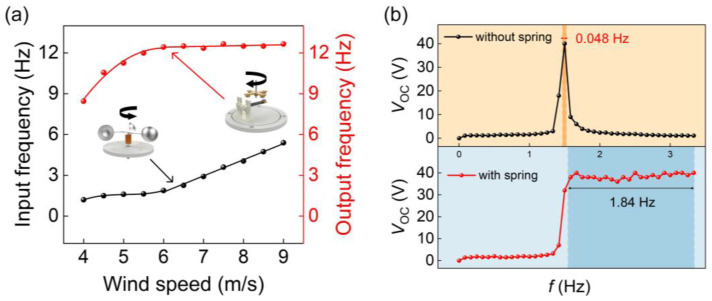
The wind-speed adaptability of A-PEH. (**a**) The correlation between input rotational frequency and output excitation frequency at various wind speeds. (**b**) The output performance of A-PEH with and without the FM module at different input rotational frequencies.

Figure 9 illustrates the input rotational frequency of the A-PEH under different wind speeds and the adaptability of the PEH with and without the FM module to the input rotational frequency. In the experiment, a blower was employed as the source of wind. The wind speed at the location of the wind cup and the input rotational frequency were measured. Analyzing the piezoelectric beam’s output voltage signal helped determine the device’s output rotational frequency. The correlation between the wind speed, input rotational frequency, and output rotational frequency is depicted in Figure 9a. The variation in the wind speed influences the rotational frequency of the input shaft and the rotational frequency of the output. When the wind speed exceeds 4 m/s, the input shaft begins to rotate, and the excitation frequency of the output is eight times the input rotational frequency. As the wind speed increases from 4 m/s to 6 m/s, both the rotational frequency of the input shaft and the rotational frequency of the output gradually rise. When the wind speed surpasses 6 m/s, the rotational speed of the input shaft shows a steady increasing trend, while the output excitation frequency remains relatively stable, indicating the wind-speed-adaptive nature of the device. From Figure 9b, it is evident that the PEH without the FM module can only maintain the sub-resonant state at an input rotational frequency of 1.473–1.521 Hz, while the A-PEH with the FM module can sustain the sub-resonant state at an input rotational frequency of 1.55–3.34 Hz. The adaptability of the A-PEH to the input rotational frequency is significantly enhanced compared to the conventional PEH. In comparison to the PEH without the FM module, the A-PEH with the FM module requires a higher excitation frequency to enter the sub-resonant state, as the FM module results in partial energy loss.

**Figure 10:**
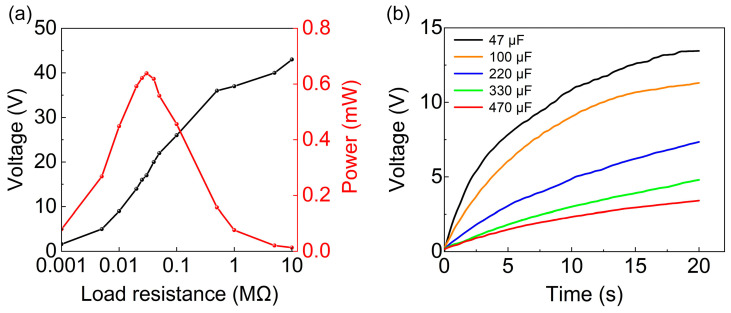
Output performance of A-PEH under loads. (**a**) Average power of A-PEH under different external resistance loads. (**b**) Charging curves for different external capacitors.

Figure 10 illustrates the output performance of the A-PEH in the presence of an external load. The output power of the A-PEH at different loads is presented in Figure 10a. Throughout the experiment, the external load of the A-PEH was systematically incremented from 1 kΩ to 10 MΩ. With an external load of 30 kΩ, the A-PEH exhibits an average output power of 0.64 mW, reaching a maximum output power of 2.5 mW. The formulas for calculating the average power *P*_avg_ and peak power *P* are expressed as follows:

(7) $$P_{\mathrm{avg}}=\frac{1}{T}\int_{0}^{T}\frac{{\left(\frac{V_{\mathrm{OC}}(t)}{2}\right)}^{2}}{R}dt$$

(8) $$P=\frac{{\left(\frac{V_{\mathrm{OC}}}{2}\right)}^{2}}{R}$$

where *V*_OC_ is the open-circuit voltage and *R* is the load.

As shown in Figure 10b, the A-PEH can charge capacitors of 47 µF, 100 µF, 220 µF, 330 µF, and 470 µF to voltages of 13.3 V, 11.2 V, 7.5 V, 5 V, and 3.3 V within 20 s.

**Figure 11:**
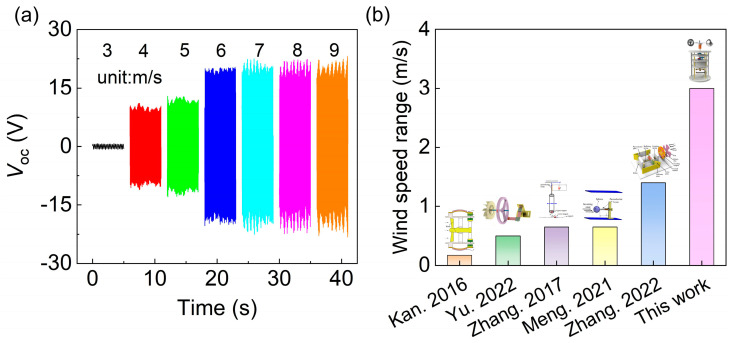
Output performance of A-PEH under various wind speed conditions and comparison of sub-resonant wind speed range with other resonant PEHs (**a**) Output performance of A-PEH at different wind speeds. (**b**) Sub-resonant wind speed range of the A-PEH with other works [23,27,28,29,30].

Figure 11 depicts the output performance of the A-PEH under various wind speed conditions and compares the operational wind speed range of the A-PEH’s sub-resonant state with that of other resonant PEHs. Figure 11a depicts the open-circuit voltage of the A-PEH at different wind speeds. The initiation wind speed for the A-PEH is 4 m/s. As the wind speed increases from 4 m/s to 6 m/s, the performance of the A-PEH gradually improves. At a wind speed of 6 m/s, the A-PEH reaches the sub-resonant state. The A-PEH consistently maintains the sub-resonant state as the wind speed exceeds 6 m/s. Figure 11b compares the wind speed range in which the A-PEH operates in the sub-resonant state with that of conventional resonant PEHs. The results indicate a significant increase in the wind speed range where the A-PEH operates in the sub-resonant state. Detailed information on relevant studies is provided in Table 1.

### 4.3. Application Demonstration

**Figure 12:**
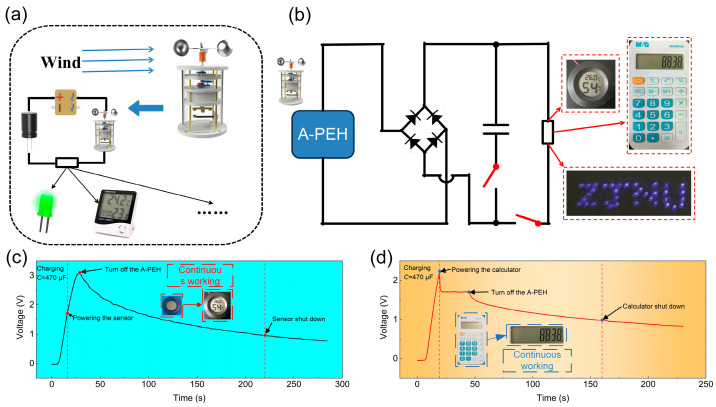
Demonstrations of A-PEH applications: (**a**) Application system powered by A-PEH. (**b**) Circuit diagram of the A-PEH application system. (**c**) Voltage variation across capacitor (470 µF) over time in powering a temperature and humidity sensor. (**d**) Voltage variation across capacitor (470 µF) over time in powering a calculator.

Figure 12a illustrates the schematic diagram of the dynamic detection system that relies on the A-PEH. The A-PEH effectively converts wind energy into electricity, providing power to a range of low-power sensors. The circuit diagram of the demonstration system is shown in Figure 12b, utilizing the A-PEH to power low-power electronics through a rectifier and a 470 µF capacitor. The A-PEH successfully powers 36 blue light-emitting diodes (Supplementary Video S3). In this experiment, the A-PEH provides a stable power supply to a calculator and temperature and humidity sensor. Figure 12c illustrates that in its sub-resonant operating state, the A-PEH efficiently charges a 470 µF capacitor to 3.1 V within a span of 21 s. After the A-PEH ceases operation, the sensor can continue working for 191 s (Supplementary Video S2). Figure 12d demonstrates that the A-PEH charges the capacitor to 2.2 V within 12 s in its sub-resonant state. The calculator can continuously operate when the A-PEH is functioning, with the capacitor voltage maintained at 1.7 V. After the A-PEH stops working, the calculator can continue to operate for 141 s (Supplementary Video S1). This underscores the capability of the A-PEH in supplying power to low-power sensors.

## 5. Conclusions

To enhance the piezoelectric energy harvester’s ability to sustain a sub-resonant state across a broader spectrum of wind velocities, this study introduces a wind-speed-adaptive resonant piezoelectric energy harvester for offshore wind energy collection. The parameters of the A-PEH were optimized through experiments, and its output performance was tested under different input rotational frequencies and various wind speeds. The conclusions of this study include the following:

i. The optimal tip mass of the piezoelectric beam is 6 g, and the optimal coil length in the spring structure is 20 mm.
ii. When the rotational frequency of the input shaft exceeds 1.57 Hz, the device’s output excitation frequency stabilizes at approximately 12.35 Hz, enabling the A-PEH to sustain a sub-resonant state. In this state, the A-PEH yields an open-circuit voltage of 40 V and an average output power of 0.64 mW.
iii. In different wind speed scenarios, the starting wind speed for the A-PEH is 4 m/s. When the wind speed surpasses 6 m/s, the A-PEH can sustain a sub-resonant state, showcasing excellent energy harvesting capacity in offshore wind environments with average wind speeds surpassing 5.5 m/s.
iv. The A-PEH has successfully demonstrated its capability to power low-power electronic devices by efficiently storing the generated electrical energy.

## Figures and Tables

**Table 1:** The output performance comparison of resonant piezoelectric energy harvesters.

Ref.	Output Voltage (V)	Average Power (mW)	Peak Power (mW)	Sub-Resonant Wind Speed Range (m/s)
Kan. et al. [23]	37.2	/	/	5.8–5.97
Yu. et al. [29]	36.8	/	0.563	11.9–12.4
Zhang. et al. [27]	/	0.15	/	2.9–3.55
Meng. et al. [28]	14	0.19	/	5.1–5.75
Zhang. et al. [30]	37.6	/	2.13	8.7–10.1
This work	20	0.64	2.5	6–9

## Data Availability

Data are contained within the article.

## Supplementary Materials

The following supporting information can be downloaded at: https://www.mdpi.com/article/10.3390/s24051371/s1, Video S1: Calculator is powered by A-PEH; Video S2: Sensor is powered by A-PEH; Video S3: LEDs are powered by A-PEH.
